# Efficacy of intermittent topical 5-fluorouracil 5% and oral nicotinamide in the skin field cancerization: a randomized clinical trial^☆^^☆☆^

**DOI:** 10.1016/j.abd.2020.09.012

**Published:** 2021-09-10

**Authors:** Eliane Roio Ferreira, Anna Carolina Miola, Thania Rios Rossi Lima, Juliano Vilaverde Schmitt, Luciana Patricia Fernandes Abbade, Hélio Amante Miot

**Affiliations:** Department of Dermatology, Faculty of Medicine, Universidade Estadual Paulista, São Paulo, SP, Brazil

Dear Editor,

Actinic keratosis (AK) is the fourth most commonly diagnosed dermatosis in Brazilian dermatology consultations and the most frequent diagnosis in patients over 65 years of age.^1^ The risk of malignancy of a single AK is low; however, multiple AKs in the same patient multiply the individual risk, which, added to the impossibility of determining which lesion will become malignant, makes the treatment and clinical follow-up of all AKs essential.^2^

The presence of more than one AK in the same area clinically characterizes active skin field cancerization (SFC).^3^ Recently, SFC stabilization strategies have been studied, aiming at preventing the incidence of skin tumors, their recurrence, or the evolution of existing lesions.

5-Fluorouracil (5FU) is a topical chemotherapeutic agent with excellent response, reducing AK counts up to 80%, and stabilizing SFC. However, side effects caused by its daily use can lead to poor adherence and a poor outcome.^4^ Despite the description of several therapeutic regimens, its intermittent use for SFC stabilization has not been adequately tested.

Nicotinamide is a B-complex vitamin that works on DNA repair, reducing the effects of skin immunosuppression caused by ultraviolet radiation (UVR), modulating the production of inflammatory cytokines, and restoring cell energy levels after exposure to UVR. Moreover, oral nicotinamide seems to have a photoprotective effect in humans, reducing AK count and the incidence of skin neoplasms in high-risk patients.^5^

An open, randomized, comparative, factorial, self-controlled, double-blind (for nicotinamide) clinical trial was carried out, in which 36 patients whose forearms had three to ten AKs each, were randomized into two groups. One group received 500 mg of oral nicotinamide every 12 hours for 120 days and the other group received a placebo at the same dose. Their forearms were subsequently randomized to receive topical 5FU in the evening, three times a week, or sunscreen with a sun protection factor (SPF) of 30 three times a day. The patients were clinically evaluated for AK counts in a standardized area of ​​the forearms, and the forearm photoaging scale (FPS), which assesses the forearms regarding photoaging aspects, such as wrinkles, melanoses, visible purpura, elastosis, and stellar scars, associated with the presence of AKs.^6^

Additionally, patients were submitted to a biopsy in the central region of the forearm, in the skin without clinically evident AKs, to evaluate epithelial dysplasia based on KIN (Keratinocyte Intraepithelial Neoplasia) and immunohistochemical analysis of p53 and Ki67 markers, at enrollment and after 120 days. The primary outcome was complete clearance of AK and the secondary ones were partial clearance (> 50%) and reduced FPS, KIN and p53, and Ki67 expression.

The analysis unit of this study was each forearm. The results were analyzed by intention to treat, and dropouts were imputed using the mixed model. Variables were compared according to time and the groups using a (multilevel) linear mixed-effects model with a robust covariance matrix. The post-hoc analysis was performed using Sidak’s sequential procedure. Statistical significance was set at p < 0.05.

The patients’ demographic data are shown in Table 1. Of the 36 analyzed patients, three were dropouts: one due to death (unrelated to the study interventions), one due to improvement in the lesions, and one due to an adverse effect of the 5FU.

**Table 1:** Demographic and clinical data of study participants.

Variables		Values
Age (years), mean (SD)		71.6 (8.6)
Sex, n (%)	Male	14 (39)
	Female	22 (61)
Fitzpatrick phototype, n (%)	I	13 (36)
	II	21 (58)
	III	2 (6)
Chronic sun exposure, n (%)		34 (94)
AK count, median (p25–p75) in each forearm		6 (5–8)

Table 2 shows the main clinical and histopathological results of the study. Improvement in AK count and photoaging scale were greater with 5FU when compared to sunscreen use, with no difference between groups in terms of total AK clearance (Fig. 1). Although nicotinamide did not provide additional clinical improvement compared to the use of 5FU or sunscreen, there was a significant reduction in Ki67 expression in the nicotinamide group compared to the other groups. There was no difference between nicotinamide and 5FU use in reducing p53 expression or improving the KIN score. Table 3 shows the reported adverse events. Only one participant had severe erythema secondary to 5FU, with consequent treatment discontinuation.

**Table 2:** Main clinical and histopathological outcomes.

	Nicotinamide		Placebo	
	5FU (n = 18)	FPS (n = 18)	5FU (n = 18)	FPS (n = 18)
**T0**				
FPS	99 (90–104)	100 (86–103)	99 (81–99)	97 (81–103)
**AK count**^d^	7 (5–8)	7 (5–9)	6.5 (5–8)	5 (5–7)
**p53**^d^	4.3 (1.2–9.5)	10.5 (3.2–12.7)	4.4 (2.4–5.3)	4.8 (2.6–7.6)
**Ki67**^d^	7.1 (4.0–8.1)	7.75 (4.9–10.3)	6.76 (4.5–8.5)	5.9 (5.0–9.0)
**KIN**^e^				
0	0 (0.0)	2 (11.1)	0 (0.0)	2 (11.1)
I	9 (50.0)	6 (33.3)	7 (38.9)	7 (38.9)
II	9 (50.0)	10 (55.6)	11 (61.1)	9 (50.0)
**T120**				
FPS^d^	90 (74–100)^a^, ^b^	94 (75–97)^a^	78 (63–95)^a^, ^b^	89 (74–95)^a^
**AK count**^d^	2 (1–4)^a^, ^b^	3.5 (2–7)^a^	1.5 (1–3)^a^, ^b^	2 (1–6)^a^
**p53**^d^	4.7 (3.8–14.4)	4.8 (3.8–9.6)	6.5 (4.5–9.0)	5.4 (3.8–8.0)
**Ki67**^d^	4.3 (3.5–6.7)^a^, ^c^	4.4 (3.1–7.1)^a^, ^c^	6.7 (5.1–7.6)^a^	6.5 (4.5–7.5)^a^
**KIN**^e^				
0	7 (38.9)^a^	6 (33.3)^a^	6 (33.3)^a^	4 (22.2)^a^
I	9 (50.0)	7 (38.9)	8 (44.4)	8 (44.4)
II	2 (11.1)	5 (27.8)	4 (22.2)	6 (33.3)
**Total AK clearance**^e^	4 (23.5)	2 (11.8)	4 (26.7)	3 (20)
**Partial AK clearance**^e^	15 (83.3)^b^	10 (55.5)	13 (72.2)^b^	11 (61.1)

a p < 0.05 (T0 *vs*. T120).

b p < 0.05 (5-FU *vs*. FPS).

c p < 0,05 (nicotinamide *vs.* placebo).

d median (p25–p75).

e n (%).

**Figure 1:**
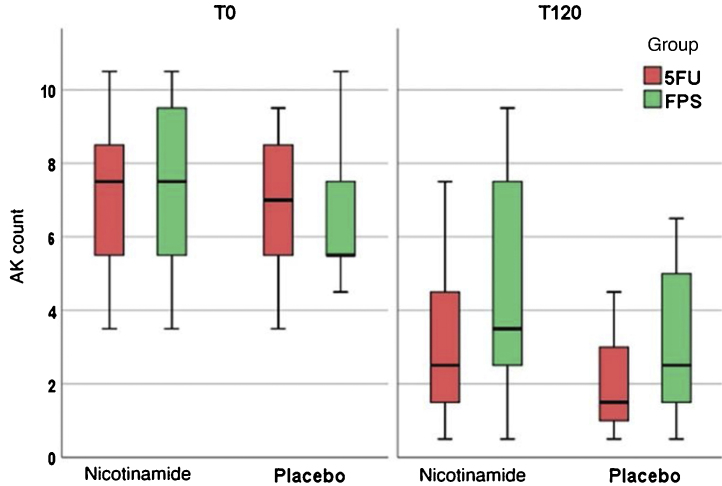
Actinic keratoses count at T0 and T120 for the oral nicotinamide, placebo, 5FU, and topical sunscreen groups: improvement in all groups with time, mainly in the group using topical 5FU.

**Table 3:** Adverse events after 15 days of treatment.

	Nicotinamide (n = 18 patients)
**Nausea**	1 (5%)
**Epigastric pain**	1 (5%)
**Vomiting**	0 (-)
**Headache**	0 (-)
**Skin flushing**	0 (-)

	5-FU (n = 36 forearms)
**Erythema**	18 (50%)
**Pain**	3 (8%)
**Edema**	1 (2%)
**Ulcerations**	1 (2%)

Previous studies with topical use of 5FU showed a greater reduction in AK count in the groups that used daily regimens, albeit with greater skin irritation and adverse effects when compared to intermittent use. By the way, 5FU was the only treatment for SFC that showed a reduction in the incidence of malignancies.^7^ Intermittent, twice-daily, weekly use led to a 66% reduction in AK count after 12 weeks, showing that it can improve adherence and tolerability, promoting fewer local adverse effects.^8^

There were predictable and controllable adverse events in the 5FU group of mild or moderate intensity, which did not cause treatment discontinuation. Only one patient developed a generalized rash after local 5FU use, leading to treatment discontinuation and highlighting the significant individual variability regarding the susceptibility to 5FU, of which toxicity has been reported in cases of dihydropyrimidine dehydrogenase deficiency or with low levels of thymidylate synthase.^9^

Previous studies using oral nicotinamide have shown reduction in AK count in high-risk patients. In this study, neither clinical improvement when compared to placebo nor an additive effect to the use of 5FU was identified. It is worth noting, however, that the use of nicotinamide in the present study lasted for 120 days, whereas in the largest previous study the drug was used, on average, for approximately 12 months.^5^ However, despite the difference in treatment time and the larger number of participants (n = 386), the reduction in AK count was only 13% after 12 months of treatment.^5^

Phototype variability, a diet poor in vitamin B3, lower hepatic metabolism rate, and younger age may be associated with different clinical responses to oral nicotinamide. The studied topography (e.g., face, upper limbs or scalp) and different degrees of AK may also justify the variability in the response to SFC treatments.

There was a change in Ki67 expression with both treatments, with a significant difference in the nicotinamide group. Thus, although there is no additional clinical response of nicotinamide to the treatments implemented in this study, there is evidence of a reduction in cellular proliferation.^10^ Longer nicotinamide regimens might lead to more robust clinical outcomes.

The present study has some limitations: it is monocentric, with limited follow-up and includes elderly participants from a public institution, which minimizes the generalization of the results. Moreover, a topical 5FU dose that is different from the usual one was used, which may also make it difficult to compare the results with other previous studies.

In conclusion, the efficacy of the treatment of SFC with topical 5FU, using an intermittent regimen for 120 days was evidenced. The present study results should stimulate clinical trials with topical 5FU using an intermittent regimen to explore its role in SFC stabilization, reducing the incidence of AKs and skin neoplasms.

## Financial support

None declared.

## Authors' contributions

Eliane Roio Ferreira: Design and planning of the study; collection, analysis, and interpretation of data; critical review of the literature; drafting and editing of the manuscript; approval of the final version of the manuscript.

Anna Carolina Miola: Collection, analysis, and interpretation of data; critical review of the literature; drafting and editing of the manuscript; critical review of the manuscript; approval of the final version of the manuscript.

Thania Rios Rossi Lima: Collection, analysis, and interpretation of data; critical review of the literature; critical review of the manuscript; approval of the final version of the manuscript.

Juliano Vilaverde Schmitt: Design and planning of the study; critical review of the literature; critical review of the manuscript; approval of the final version of the manuscript.

Luciana Patricia Fernandes Abbade: Design and planning of the study; critical review of the literature; critical review of the manuscript; approval of the final version of the manuscript.

Hélio Amante Miot: Design and planning of the study; effective participation in research orientation; statistical analysis; critical review of the literature; critical review of the manuscript; approval of the final version of the manuscript.

## Conflicts of interest

None declared.
